# Universal coverage and utilization of free long-lasting insecticidal nets for malaria prevention in Ghana: a cross-sectional study

**DOI:** 10.3389/fpubh.2023.1140604

**Published:** 2023-05-25

**Authors:** Seth Kwaku Afagbedzi, Yakubu Alhassan, Ernest Kenu, Keziah Malm, Delia Akosua Benewaah Bandoh, Nana Yaw Peprah, Otubea Owusu Ansah, Chris Guure

**Affiliations:** ^1^Department of Biostatistics, School of Public Health, University of Ghana, Accra, Ghana; ^2^Department of Epidemiology and Disease Control, School of Public Health, University of Ghana, Accra, Ghana; ^3^National Malaria Control Programme, Ghana Health Service, Accra, Ghana; ^4^Ghana Field Epidemiology and Laboratory Training Programme, Accra, Ghana; ^5^Department of Global Health and Population, Harvard T.H. Chan School of Public Health, Boston, MA, United States

**Keywords:** universal coverage, utilization of insecticidal nets, long-lasting insecticide nets, mosquito nets, point mass distribution, malaria prevention

## Abstract

**Background:**

Malaria continues to be one of the leading causes of mortality and morbidity, especially among children and pregnant women. The use of Long-Lasting Insecticide Nets (LLINs) has been recognized and prioritized as a major intervention for malaria prevention in Ghana. This study aims to establish the factors influencing the universal coverage and utilization of LLINs in Ghana.

**Methods:**

The data used for this study was from a cross-sectional survey carried out to assess LLINs ownership and use in 9 out of the 10 old regions of Ghana from October 2018 to February 2019 where free LLIN distribution interventions were implemented. The EPI “30 × 7” cluster sampling method (three-stage sampling design) was modified to “15 × 14” and used for the study. A total of 9,977 households were interviewed from 42 districts. Descriptive statistics using percentages as well as tests of associations such as Pearson Chi-square and the magnitude of the associations using simple and multivariable logistic regression were implemented.

**Results:**

Of the 9,977 households in the study, 88.0% of them owned at least one LLIN, universal coverage was 75.6%, while utilization was 65.6% among households with at least one LLIN. In the rural and urban areas, 90.8% and 83.2% of households, respectively, owned at least one LLIN. The was a 44% increase in universal coverage of LLINs in rural areas compared to urban areas (AOR: 1.44, 95% CI: 1.02–2.02). There were 29 higher odds of households being universally covered if they received LLIN from the PMD (AOR: 29.43, 95% CI: 24.21–35.79). Households with under-five children were 40% more likely to utilize LLIN (AOR: 1.40, 95% CI: 1.26–1.56). Respondents with universal coverage of LLIN had 25% increased odds of using nets (AOR: 1.25 95% CI: 1.06–1.48). Rural dwelling influences LLIN utilization, thus there was about 4-fold increase in household utilization of LLINs in rural areas compared to urban areas (AOR: 3.78, 95% CI: 2.73–5.24). Household size of more than 2 has high odds of LLINs utilization and awareness of the benefit of LLINs (AOR: 1.42, 95% CI: 1.18–1.71).

**Conclusion:**

About nine in 10 households in Ghana have access at least to one LLIN, three-quarters had universal coverage, and over two-thirds of households with access used LLIN. The predictors of universal coverage included region of residence, rural dwellers, and PMD campaign, while households with child under-five, in rural areas, and with universal coverage were positively associated with utilization.

## Introduction

The estimated number of malaria cases recorded worldwide in 2021 was estimated to be 247 million, which was 2 and 15 million cases more than 2020 and 2019 figures, respectively. The total number of deaths from malaria globally in 2021 was estimated at 619,000 compared to 625,000 deaths in 2020 and 568,000 deaths in 2019 (1–3). World Health Organization African region accounted for 95% of the global malaria cases in 2021. The percentage of global malaria deaths accounted for by African regions alone in 2021 is estimated at 96% (1–3).

Long-lasting insecticide net (LLIN) is one of the core interventions recommended by the WHO for malaria vector control in SSA and has been responsible for an estimated two-thirds of the reduction in the global burden of malaria in recent years (4). In 2021, 47% of the world population slept under an LLIN, an increase from 3% in 2000 (3). The percentage of the sub-Saharan Africa population with at least one ITN increased from 5% in 2000 to 68% in 2021. During the same period, the percentage of the population with access to an ITN within their household increased from 3 to 54% (3).

Malaria is endemic in Ghana, which means that about 31 million residents are susceptible to malaria infection (4–6). Ghana contributed 2% of the global malaria morbidity and mortality in 2021 (3). Incidence of malaria accounted for 20.3% of causes of outpatient morbidity with pregnant women, children under 5 years, and immuno-compromised being the most vulnerable group (5, 7, 8). The Ghana Health Service through its agency, National Malaria Control Programme, over the years adopted a number of strategic plans to combat malaria, some of which include Roll Back Malaria (RBM) initiative in 1999 that emphasized the strengthening of health services through multi-and inter-sectoral partnerships and making treatment and prevention strategies more widely available; in the year 2000, the first National Malaria Strategic Plan (2000–2010) was developed with the goal to reduce malaria specific morbidity and mortality by 50% by the year 2010; and most recently the 2015–2020 Ghana Malaria Strategic Plan which aimed to reduce malaria burden in the country by 75% (6, 8).

The use of Long-Lasting Insecticide Nets (LLINs) has been recognized and prioritized as a major intervention for malaria prevention by the various strategies adopted in Ghana. The President Malaria Initiative (PMI) continues to support Ghana’s LLINs strategy aimed at achieving universal coverage of LLINs through complementary distribution channels: mass campaign distribution and continuous distribution. In line with the Ghana Malaria Strategic plan (2014–2020), the goal of the mass LLIN distribution campaign is to protect at least 80% of the population at risk with effective malaria prevention interventions. The GHS through National Malaria Control Programme (NMCP) implements Point Mass Distribution (PMD) of LLINs campaigns as one of its strategies. NMCP has implemented three rounds of mass LLIN distribution campaigns since 2010 and the last round was completed in December 2018. With support from partners such as United Nations International Children’s Emergency Fund (UNICEF), Department for International Development (DFID), Global Fund to Fight AIDS, Tuberculosis and Malaria (GFATM) and others between December 2010 and October 2012. National Malaria Control Programme has distributed nearly 12.5 million free LLINs through the PMD campaign in all the then 10 regions of Ghana (9). The second round of PMD LLINs campaign, which ended in the year 2016, saw the distribution of a total of 4,888 LLINs in Greater Accra, Northern, Upper East, Upper West, and Eastern Regions. During the first half of 2016, the PMD of LLIN was conducted in Upper West and Northern regions and a total of 2,457,872 LLINs were distributed (10). The third round of mass distribution of LLINs ended in 2018, with about 15.5 million LLINs (11) distributed in 194 districts in nine of the country’s 10 regions.

Ghana’s 2010–2012 mass LLIN distribution campaign saw the distribution of approximately 12.8 million LLINs (9). Thereafter, the country has been struggling to reach and sustain universal coverage and continuous use of LLINs despite so much commitment by the Ghana Health Service (GHS) and its agency NMCP, implementing different strategies (12). The RBM Partnership’s scaling-up for impact strategy was implemented in Ghana to reduce malaria-related mortality by about 75% from the year 2000 (base) by 2015. This therefore pushed Ghana to set specific targets to achieve this level of impact, which included 100% of households owning at least one LLIN, one LLIN available per two persons, 80% of the general population sleeping under an LLIN and 85% of children under 5 years and pregnant women sleeping under LLIN (4, 12). Ghana as a malaria-endemic country, subscribed to the WHO Global Technical Strategy for Malaria 2016–2030 (WHO-GTS) which has one of its goals, universal access to malaria prevention with the recommendation of universal coverage of the population at risk with LLINs (10). Alongside the continuous distribution of LLINs to pregnant women and children under-5 in schools and health facilities, Ghana is also implementing a mass distribution of free LLINs every 3 years since 2012, with the latest conducted in 2018.

Despite the progress made in the last decade to increase the coverage of LLINs distribution, Ghana has not been able to achieve the target of 100% LLINs ownership and usage of 80% in the general population. Literature has shown that LLINs ownership is usually higher than usage. A study conducted in the Hohoe Municipality among mothers with children under five showed that LLINs ownership was 81.3%, and usage was 66.4% (13). Another study conducted among 300 pregnant women seeking antenatal care in an urban hospital in the Ashanti region, reported a net ownership of 78 and 61% usage (14). This study was undertaken to determine the factors that influence the universal coverage and utilization of LLINs following the recent PMD distribution campaign in 2018, which covered nine out of the old 10 regions in Ghana.

## Methods

### Study design and setting

The study was a cross-sectional survey to assess LLINs ownership and use in Ghana. The survey employed a stratified three-stage sample design with the aim of estimating key malaria indicators at the national level. The first stage involved the selection of districts followed by clusters made up of enumeration areas, which was drawn from the 2010 census data. At the third stage, a sampling frame made up of all households to allow for equal chances of selection and to make the data obtained nationally representative was adopted. Households to be included in the survey were randomly selected from the sampling frame. Quantitative data was collected from October 2018 to February 2019.

### Study population and sampling methods

The study population was head of households, pregnant women, mothers, and caretakers of children less than 5 years. The study was conducted in nine (9) out of the 10 traditional regions in Ghana (before the creation of the new regions) where interventions (PMD) were implemented, these including Ashanti, Brong Ahafo, Central, Eastern, Greater Accra, Northern, Upper East, Volta, and Western Regions. Upper West region was excluded from the sampling frame because interventions were not implemented there.

The sampling frame was the entire eligible 205 districts in the 9 regions of Ghana. The sampling frame excluded the population living in collective housing units such as hotels, hospitals, work camps, prisons, or boarding schools. The sample was stratified to provide adequate representation of urban and rural areas, as well as all eligible regions and districts.

**Figure 1:**
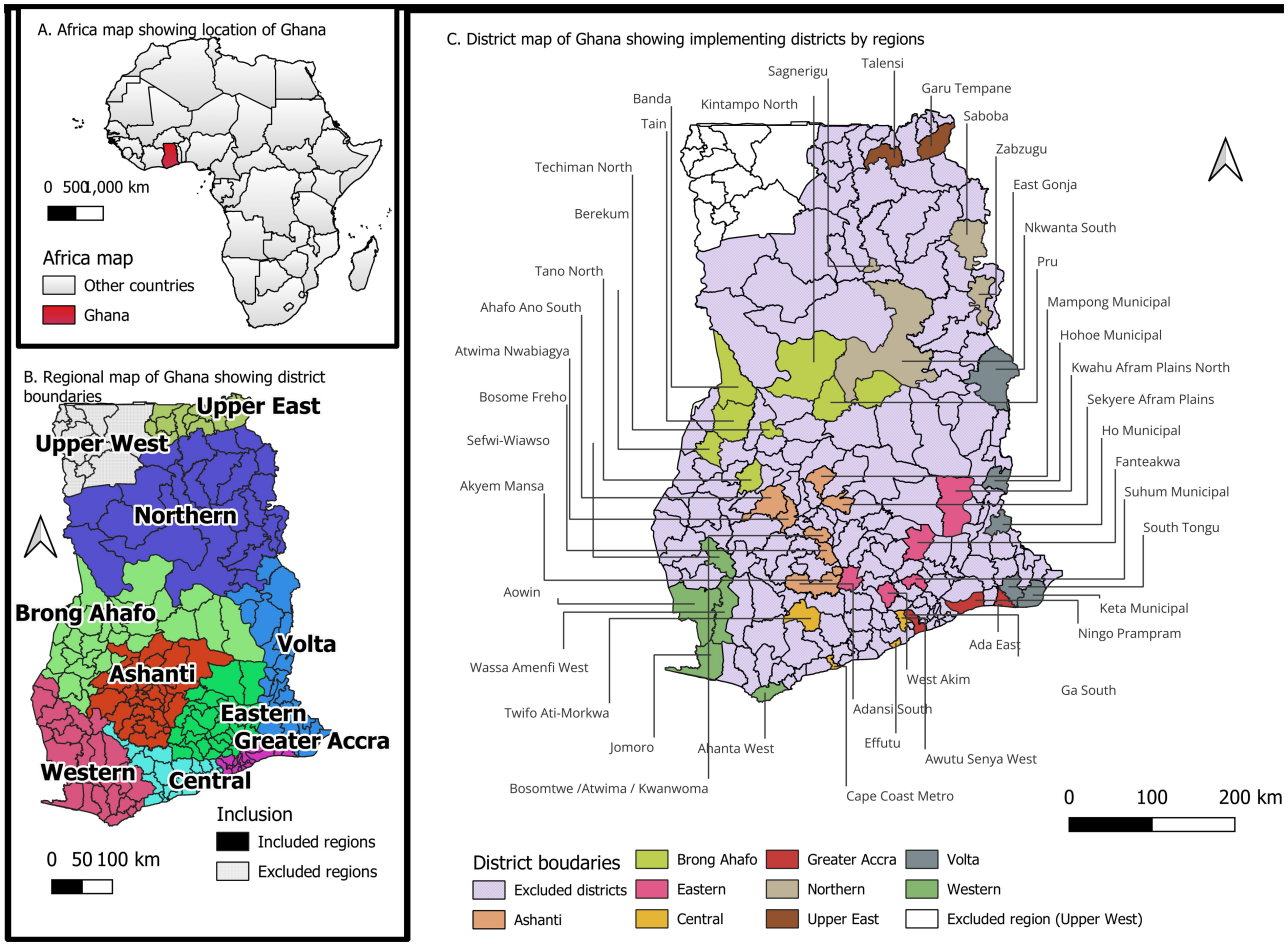
Map of Africa showing the location of Ghana **(A)**, Map of Ghana showing the implementing regions **(B)** and the implementing districts **(C)**.

The first stage of the sampling was to select districts from each region randomly proportionate to the number of districts in the region. A total of 42 districts were selected from 205 districts in the 9 regions of Ghana, representing 20.5% of the eligible districts. In effect, six (7) districts were selected from the Ashanti region, six (6) from Brong Ahafo, and five (5) each from Eastern, Volta, and Western Regions, respectively. Four (4) districts each were also selected from Central, Northern Regions and Greater Accra region, and two (2) from Upper East. The second stage was the selection of primary sampling units (PSUs), which are also called clusters based on the list of enumeration areas (EAs) created in the 2010 Population and Housing Census. The third stage of selection was at the household level in each cluster. Figure 1A shows the location of Ghana on Africa map, Figure 1B shows the implementing regions on the map of Ghana and Figure 1C shows the locations of the implementing districts in the country.

**Figure 2:**
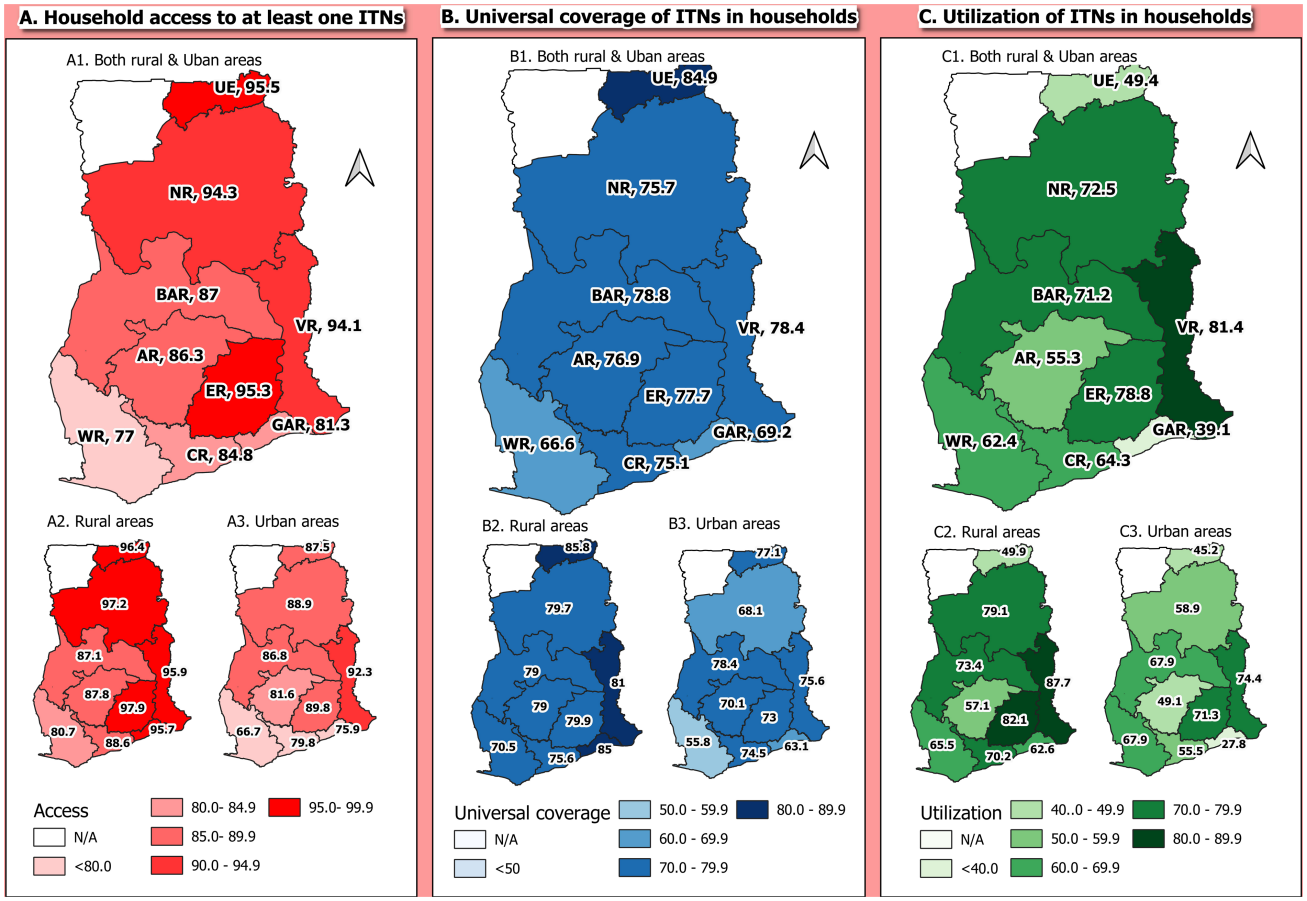
Maps of the regional and rural–urban distribution of access **(A)**, universal coverage **(B)**, and utilization **(C)** of LLINs by households.

### Selection of communities, houses, and households

The EPI “30 × 7” cluster sampling method was modified to “15 × 14” and used for the quantitative study. Thus, 15 clusters from each identified district were selected. In each cluster, 14 heads of households were randomly selected and interviewed. This gave a total of 210 households interviewed for each district and 8,820 households for the entire study. The modification allowed more samples to be collected within cluster especially when the homogeneity within cluster is likely to be high. Logistics was also a factor to this modification as it was relatively more expensive to reach a high number of clusters with smaller number of households than to reach a smaller number of clusters with high number of household, especially when similarities across districts within region is high.

In each EA, the base was identified. Field workers spanned a pencil at the EA base and followed where the tip of the pencil pointed, and the clockwise direction followed for the selection of households from the various houses in that direction till all 14 households were selected. At the household level, the head of the household was interviewed. In the absence of the household head, all eligible representatives of the household head were listed alphabetically by their given first names. The first person on the ordered list was interviewed in that household. For this study, a household was defined as a group of people who live together and eat from the same pot. Polygamous families were considered as one unit if they have one household head.

### Training

A three (3) day training on how to administer the questionnaires for the quantitative aspect of the study with a mobile application software known as Research Electronic Data Capture (REDCap) was organized for 30 research assistants from 22nd to 24th October 2018. Pre-testing of the questionnaires was done in Kimbu, a community in Greater Accra region. A total of 12 research assistants, comprising two (2) supervisors and 10 data collectors were assigned for data collection in each region. Research assistances were taken through both the English version of the questionnaire and the local dialect.

### Data collection and management

Structured questionnaire with both closed and opened ended questions was used for this study. In each EA, community entry was done with appropriate authorities. Data collectors established a rapport with household heads and administered informed consent before interviews were conducted. A questionnaire which covered the entire point mass distribution (PMD) process (registration and code card distribution, net redemption, and Social and Behavioral Change Communication, SBCC) was administered in each household. Hanged nets and any evidence of nets used during the previous night and unused nets from the PMD available in the household were observed during the interview. Data collection was done with an online data collection management system called Research Electronic Data Capture System (REDCap). Therefore, data collection, tracking, and management were done in real time. At the close of each day, the data collected was synced into the REDCap server. Daily, the uploaded data was cleaned, reconciled with data collected from the field. A back-up copy of the data was saved each day in another cloud to prevent data loss.

### Ethics approval

This was a secondary data obtained from the National Malaria Control Programme. The survey was a subsection (implementation research findings of the malaria program) of the main Point Mass Distribution exercise, and it was approved by the Ghana Health Service.

### Study variables

#### Outcomes

There are two main outcome variables for this study, these are, *Universal Coverage* defined as households with at least one LLIN per every two household members and the other outcome is *LLIN utilization,* defined as the use of at least one LLIN by a household the night prior to the survey among households with at least one LLIN.

#### Predictors

Important predictors of the outcome variables were categorized into household characteristics (Region, Residence, Household size, Household has, child under-5, Household has a pregnant woman, received, net from PMD campaign, Number of nets in household) and respondent characteristics (Household respondents, Respondent’s age, Marital status, Highest education, Religion, Employment status, Aware of benefits of LLINs, Aware of key facts of LLINs, Aware of continuous use and care for LLINs).

#### Data analysis

Analyses of the data were done using both descriptive and inferential statistics. In the descriptive part, frequencies, proportions, or percentages and charts were employed. These were carried out and presented according to the outcomes and further delineated into the levels of each of the predictors. A continuous variable such as age of the respondent was categorized into two (18–49 and >49 years) levels for ease of the analysis and interpretation. The Choropleth map was used to display access to, universal coverage and utilization of LLINs by region and further by rural and urban areas within regions.

Further analyses were done using the simple logistic regression model and Pearson Chi-square test to establish associations or relationships between the two main outcomes (universal coverage and utilization) of interest with each of the predictor variables. Multivariable logistic regression was then implemented after variables were selected from the bivariate analysis due to their significance with a type-I error of 5%. Variables such as respondents’ age, employment status, and religion were included due to their relevance as observed from literature. In addition, two variable interaction effects were examined for combinations of the predictors and none except residence and region interaction was significant. This was therefore included in the final multivariable logistic regression model to obtain adjusted odds ratios.

Analysis was conducted using Stata IC version 16 (Stata Corp., College Station, TX, United States). All statistical analyses were considered significant at a predefined alpha value of 0.05.

## Results

### Descriptive characteristics of households in the study

A total of 9,977 households were interviewed. The Ashanti region (16.6%) was the most represented while the Upper East region (4.7%) was least represented. About two-thirds (62.6%) of the households interviewed were in rural areas. The median household size was 5 members (IQR: 3 to 6 members) with approximately half (51.3%) of the households having a child below 5 years and while 6.9% had a pregnant woman. Majority (86.2%) of the households had received LLINs from the PMD campaign (Table 1).

**Table 1:** Descriptive characteristics of households and household respondents.

Variables and categories	Frequency (%)	*N* = 9,977
**Region**	
Greater Accra	960 (9.6)
Ashanti	1,661 (16.6)
Eastern	1,226 (12.3)
Central	937 (9.4)
Volta	1,193 (12.0)
Brong Ahafo	1,398 (14.0)
Western	1,144 (11.5)
Northern	988 (9.9)
Upper East	470 (4.7)
**Residence**	
Rural	6,248 (62.6)
Urban	3,729 (37.4)
Household size, median (IQR)	5 (3, 6)
**Household size**	
1–2 members	1,677 (16.8)
3–5 members	4,726 (47.4)
6–7 members	2,183 (21.9)
8 or more	1,382 (13.9)
**Household has child under-5**^**M**^	
No	4,824 (48.7)
Yes	5,073 (51.3)
**Household has a pregnant woman**	
No	9,285 (93.1)
Yes	692 (6.9)
**Received net from PMD campaign**	
No	1,379 (13.8)
Yes	8,598 (86.2)
**Respondents’ characteristics** **Respondent household**	
Household head	4,484 (44.9)
Others	5,493 (55.1)
**Respondent’s age, median (IQR)**	41.5 (38.0, 52.0)
**Respondent’s age**^**M**^	
18-49 years	7,045 (71.0)
>49 years	2,883 (29.0)
**Sex**	
Male	2,657 (26.6)
Female	7,320 (73.4)
**Marital status**^**M**^	
Single	1,559 (15.7)
Divorced/separated	708 (7.1)
Cohabiting	315 (3.2)	Variables and categories	Frequency (%)	*N* = 9,977
Widowed	988 (9.9)
Married	6,376 (64.1)
**Highest education**^**M**^	
No formal education	3,110 (31.4)
Primary	1,518 (15.3)
JHS/JSS/Middle	3,697 (37.3)
SHS/SSS/VOC/TECH	1,157 (11.7)
Tertiary	423 (4.3)
**Religion**^**M**^	
None	271 (2.7)
Christian	7,888 (79.2)
Muslim	1,411 (14.2)
Traditional	389 (3.9)
**Employment status**^**M**^	
Unemployed	1,283 (12.9)
Employed	8,687 (87.1)
**Aware of benefits of LLINs**	
Yes	6,299 (63.1)
No	3,678 (36.9)
**Aware of key facts of LLINs**	
Yes	6,241 (62.6)
No	3,736 (37.4)
**Aware of continuous use and care for LLINs**	
Yes	4,056 (40.7)
No	5,921 (59.3)

M, Variable has missing observations.

Less than half (44.9%) of the respondents were the household head. The median age of the respondents was 41.5 years (IQR: 38 to 52 years). Majority (73.4%) of the respondents were females. Majority (64.1%) were also married. About a third had no formal education (31.4%) While 4.3% had tertiary education. Majority (79.2%) were Christians (79.2%) or were employed (87.1%). Majority were also aware of the benefits of LLINs (63.1%), the key facts of LLINs (62.6%), while less than half (40.7%) were aware of the continuous use and care of LLINs (Table 1).

### LLINs ownership among households interviewed

Of the 9,977 households in the study, 88.0% (8,777/9,977) of the households owned at least one LLIN. 95.5% (449/470), 95.3% (1,168/1,226), and 94.3% (932/988) of households from the Upper East, Eastern, and Northern regions, respectively, owned at least one net, the highest. About 77.0% (881/1,144) of the households in the Western region owned at least one LLIN being the least. In rural areas, 90.8% (5,675/6,248) of the households owned at least one LLIN, while 83.2% (3,102/3,729) of households in the urban areas also owned at least one LLIN. Household access to at least one LLIN was higher in the rural areas than the urban areas for each of the 9 regions. Figure 1A shows the distribution of access to LLINs among households across regions and also rural–urban distributions (Figure 1).

### Universal coverage of LLINs among households

Out of the 9,977 total households interviewed, about three-quarters (75.6%, n = 7542/9977) had universal coverage of LLINs. Among the nine regions, the universal coverage of LLINs was highest in the Upper East region (84.9%) and lowest in the western region (66.6%). Households in rural areas (78.7%) had a higher percentage of universal coverage of LLINs compared to households in urban areas (70.4%). Universal coverage of LLINs was highest in the rural areas than the urban areas in all 9 regions. Figure 1B shows the distribution of the universal coverage of LLINs among households across regions and also rural–urban distributions (Figure 1).

### Utilization of LLINs among households with access to at least one LLINs

About 8,777 households have access to at least one LLINs, out of which two-thirds (65.6%) had at least one member of the household sleeping under LLINs the night before the survey. Among the nine regions, household utilization of LLINs was highest in the Volta region (81.4%) and lowest in the Greater Accra region (39.1%). Utilization of LLINs was higher among households in rural areas (70.2%) compared to households in urban areas (57.1%). Utilization of LLIN was also higher in rural areas compared to the urban areas in each of the 9 regions. Figure 1C shows the distribution of the utilization of LLINs among households across regions and also rural–urban distributions (Figure 1).

### Bivariate analysis of factors associated with universal coverage of LLINs

The Pearson’s Chi-square test showed that region of residence (χ^2^ = 110.6, *p* < 0.001), area of residence (χ^2^ = 86.4, *p* < 0.001), household size (χ^2^ = 160.7, *p* < 0.001), children under-5 years in household (χ^2^ = 11.4, *p* < 0.01), and household receiving LLINs from recent PMD campaign (χ^2^ = 2,700, *p* < 0.001) were household characteristics that were significantly associated with universal coverage of LLINs. The statistically significant respondents’ characteristics associated with universal coverage of LLINs were household head (χ^2^ = 3.9, *p* < 0.05), the age of the respondent (χ^2^ = 5.3, *p* < 0.05), marital status of the respondent (χ^2^ = 23.2, *p* < 0.001), and the highest education of the respondent (χ^2^ = 13.3, *p* < 0.05). The awareness of respondents on the benefits of LLINs (χ^2^ = 838.5, *p* < 0.001), key facts about LLINs (χ^2^ = 874.9, *p* < 0.001), and the continuous use and care for LLINs (χ^2^ = 177.7, *p* < 0.001) were also significantly associated with universal coverage of LLINs in households (Table 2).

**Table 2:** Bivariate analysis of factors associated with universal coverage of LLINs and utilization of LLINs.

Variables	Universal coverage of LLINs			Household utilization of LLINs			Total	Covered	χ^2^ value of *p*	Total	Use	χ^2^, value of *p*	*N*	*n* (%)	*N*	*n* (%)
Total	9,977	7,542(75.6)		8,777	5,756(65.6)	
**Household characteristics**						
Region			110.6^***^			617.4^***^
Greater Accra	960	664(69.2)		780	305(39.1)	
Ashanti	1,661	1,277(76.9)		1,433	792(55.3)	
Eastern	1,226	952(77.7)		1,168	920(78.8)	
Central	937	704(75.1)		795	511(64.3)	
Volta	1,193	935(78.4)		1,123	914(81.4)	
Brong Ahafo	1,398	1,101(78.8)		1,216	866(71.2)	
Western	1,144	762(66.6)		881	550(62.4)	
Northern	988	748(75.7)		932	676(72.5)	
Upper East	470	399(84.9)		449	222(49.4)	
Residence			86.4^***^			152.0^***^
Rural	6,248	4,916(78.7)		5,675	3,984(70.2)	
Urban	3,729	2,626(70.4)		3,102	1772(57.1)	
Household size			160.7^***^			43.7^***^
1–2 members	1,677	1,380(82.3)		1,353	785(58.0)	
3–5 members	4,726	3,651(77.3)		4,193	2,775(66.2)	
6–7 members	2,183	1,628(74.6)		1964	1,339(68.2)	
8 or more	1,382	876(63.4)		1,260	855(67.9)	
Household has child under-5^M^			11.4^**^			105.5^***^
No	4,824	3,717(77.1)		4,075	2,445(60.0)	
Yes	5,073	3,761(74.1)		4,634	3,266(70.5)	
Household has a pregnant woman			1.9			1.2
No	9,285	7,034(75.8)		8,160	5,339(65.4)	
Yes	692	508(73.4)		617	417(67.6)	
Received net from PMD campaign			2700.0^***^			48.6^***^
No	1,379	270(19.6)		598	314(52.5)	
Yes	8,598	7,272(84.6)		8,179	5,442(66.5)	
Number of nets in household						61.9^***^
One	–	–		1,211	680(56.2)	
Two	–	–		2024	1,316(65.0)	
Three	–	–		2,233	1,495(67.0)	
>Three	–	–		3,309	2,265(68.5)	
Universal coverage of LLINs						10.8^**^
Not covered	–	–		1,446	894(61.8)	
Covered	–	–		7,329	4,860(66.3)	
Respondents’ characteristics						
Respondent household			3.9^*^			1.4
Household head	4,484	3,432(76.5)		3,870	2,564(66.3)	
Others	5,493	4,110(74.8)		4,907	3,192(65.1)	
Respondent’s age ^M^			5.3^*^			3.3
18-49 years	7,045	5,283(75.0)		6,187	4,104(66.3)	
>49 years	2,883	2,225(77.2)		2,550	1,640(64.3)	
Sex			1.06			2.2
Male	2,651	1989 (74.9)		2,274	1,520(66.8)	
Female	7,295	5,553 (75.9)		6,503	4,236(65.1)	
Marital status ^M^			23.2^***^			57.2^***^
Single	1,559	1,111(71.3)		1,225	711(58.0)	
Divorced/separated	708	552(78.0)		616	386(62.7)	
Cohabiting	315	231(73.3)		271	184(67.9)	
Widowed	988	772(78.1)		890	545(61.2)	
Married	6,376	4,852(76.1)		5,749	3,914(68.1)	
Highest education ^M^			13.3^*^			18.1^**^
No formal education	3,110	2,389(76.8)		2,819	1849(65.6)	
Primary	1,518	1,141(75.2)		1,352	918(67.9)	
JHS/JSS/Middle	3,697	2,806(75.9)		3,219	2,143(66.6)	
SHS/SSS/VOC/TECH	1,157	864(74.7)		999	605(60.6)	
Tertiary	423	292(69.0)		331	204(61.6)	
Religion ^M^			4.3			23.0^***^
None	271	193(71.2)		231	161(69.7)	
Christian	7,888	5,984(75.9)		6,911	4,570(66.1)	
Muslim	1,411	1,051(74.5)		1,261	759(60.2)	
Traditional	389	298(76.6)		358	253(70.7)	
Employment status ^M^			0.6			11.1^**^
Unemployed	1,283	959(74.8)		1,117	683(61.2)	
Employed	8,687	6,579(75.7)		7,656	5,070(66.2)	
Aware of benefits of LLINs			838.5^***^			181.8^***^
Yes	6,299	5,361(85.1)		6,008	4,219(70.2)	
No	3,678	2,181(59.3)		2,769	1,537(55.5)	
Aware of key facts of LLINs			874.9^***^			149.8^***^
Yes	6,241	5,332(85.4)		5,957	4,161(69.9)	
No	3,736	2,210(59.2)		2,820	1,595(56.6)	
Aware of continuous use and care for LLINs			177.7^***^			200.0^***^
Yes	4,056	3,347(82.5)		3,740	2,764(73.9)	
No	5,921	4,195(70.9)		5,037	2,992(59.4)	

M, Variable has missing observations. *p*-value notation: ^*^p < 0.05. ^**^p < 0.01. ^***^p < 0.001.

### Bivariate analysis of factors associated with household utilization of LLINs

Pearson’s Chi-square test showed that region of residence (χ^2^ = 617.4, *p* < 0.001), area of residence (χ^2^ = 152.0, *p* < 0.001), household size (χ^2^ = 43.7, *p* < 0.001), household with children under-5 years (χ^2^ = 105.5, *p* < 0.001), household receiving LLINs from PMD’s distribution campaign (χ^2^ = 48.6, *p* < 0.001), number of LLINs in household (χ^2^ = 61.9, *p* < 0.001), and household having at least 1 net per every 2 persons (χ^2^ = 10.8, *p* < 0.01) were household characteristics significantly associated with utilization of LLINs. The marital status of the respondent (χ^2^ = 57.2, *p* < 0.001), the highest education of the respondent (χ^2^ = 18.1, *p* < 0.05), religion of the respondent (χ^2^ = 23.0, *p* < 0.001), and employment status (χ^2^ = 11.1, *p* < 0.01) were respondents characteristics significantly associated with utilization of LLINs. The awareness of respondents on the benefits of LLINs (χ^2^ = 181.8, *p* < 0.001), key facts about LLINs (χ^2^ = 149.8, *p* < 0.001) and the continuous use and care for LLINs (χ^2^ = 200.0, *p* < 0.001) were also significantly associated with universal coverage of LLINs in households (Table 2).

### Multivariable analysis of factors associated with universal coverage of LLINs

The binary logistic regression model was used to estimate both the crude and adjusted odds ratio of factors associated with universal coverage of LLINs among the households. From the adjusted model, the odds of universal coverage of LLINs among households in the Greater Accra (AOR: 2.86, 95% CI: 1.99–4.12), Ashanti (AOR: 2.03, 95% CI: 1.36–3.02), Eastern (AOR: 2.24, 95% CI: 1.50–3.36), Central (AOR: 2.24, 95% CI: 1.49–3.35), Volta (AOR: 3.02, 95% CI: 2.07–4.40), Brong Ahafo (AOR: 2.99, 95% CI: 2.03–4.40), and Northern (AOR: 1.93, 95% CI: 1.29–2.90) regions were significantly higher compared to households from the Western region. There was a 44% increase in universal coverage of LLINs in rural areas compared to urban areas (AOR: 1.44, 95% CI: 1.02–2.02).

An interaction effect between residence (rural) and region of residence showed increased universal coverage in the rural parts across all 9 regions; Greater Accra (AOR: 3.00, 95% CI: 2.14–4.21), Ashanti (AOR: 2.14, 95% CI: 1.51–3.03), Eastern (AOR: 2.19, 95% CI: 1.50–3.19), Central (AOR: 3.39, 95% CI: 2.32–4.97), Volta (AOR: 2.39, 95% CI: 1.69–3.40), Brong Ahafo (AOR: 1.44, 95% CI: 1.02–2.02), Western (AOR: 2.85, 95% CI: 1.97–4.12), Northern (AOR: 3.44, 95% CI: 2.24–5.29), and Upper East (3.00, 95% CI: 2.14–4.21; Table 3).

**Table 3:** Multivariate analysis of factors associated with universal coverage of LLINs and utilization of LLINs.

Variables	Household coverage of LLINs		Household utilization of LLINs		COR [95% CI]	AOR [95% CI]	COR [95% CI]	AOR [95% CI]
**Household characteristics**				
**Region**				
Greater Accra	1.12 [0.94–1.35]	2.86 [1.99–4.12]^***^	1.00 [reference]	1.00 [reference]
Ashanti	1.00 [reference]	1.00 [reference]	2.59 [2.12–3.15]^***^	2.59 [1.82–3.68]^***^
Eastern	1.67 [1.41–1.97]^***^	2.03 [1.36–3.02]^**^	1.92 [1.61–2.30]^***^	2.19 [1.63–2.94]^***^
Central	1.74 [1.45–2.09]^***^	2.24 [1.50–3.36]^***^	5.78 [4.73–7.06]^***^	5.02 [3.68–6.86]^***^
Volta	1.51 [1.25–1.84]^***^	2.24 [1.49–3.35]^***^	2.80 [2.28–3.44]^***^	2.90 [2.13–3.93]^***^
Brong Ahafo	1.82 [1.51–2.19]^***^	3.02 [2.07–4.40]^***^	6.81 [5.53–8.39]^***^	6.49 [4.90–8.60]^***^
Western	1.86 [1.56–2.22]^***^	2.99 [2.03–4.40]^***^	3.85 [3.19–4.66]^***^	5.01 [3.79–6.63]^***^
Northern	1.56 [1.29–1.89]^***^	1.93 [1.29–2.90]^**^	4.11 [3.36–5.04]^***^	3.52 [2.53–4.88]^***^
Upper East	2.82 [2.13–3.73]^***^	2.39 [0.93–6.11]	1.52 [1.20–1.93]^***^	1.71 [0.89–3.28]
**Residence**				
Rural	1.55 [1.41–1.70]^***^	1.44 [1.02–2.02]^*^	1.77 [1.61–1.94]^***^	3.78 [2.73–5.24]^***^
Urban	1.00 [reference]	1.00 [reference]	1.00 [reference]	1.00 [reference]
**Interaction (Region # Residence)**				
Greater Accra # Rural	–	3.00 [2.14–4.21]^***^	–	3.78 [2.73–5.24]^***^
Ashanti # Rural	–	2.14 [1.51–3.03]^***^	–	2.93 [2.32–3.70]^***^
Eastern # Rural	–	2.19 [1.50–3.19]^***^	–	8.96 [6.80–11.80]^***^
Central # Rural	–	3.39 [2.32–4.97]^***^	–	4.67 [3.51–6.23]^***^
Volta # Rural	–	2.39 [1.69–3.40]^***^	–	15.99 [11.48–22.25]^***^
Brong Ahafo # Rural	–	1.44 [1.02–2.02]^*^	–	5.97 [4.59–7.75]^***^
Western # Rural	–	2.85 [1.97–4.12]^***^	–	4.49 [3.46–5.82]^***^
Northern # Rural	–	3.44 [2.24–5.29]^***^	–	8.13 [6.05–10.92]^***^
Upper East # Rural	–	3.00 [2.14–4.21]^***^	–	2.15 [1.61–2.86]^***^
**Household size**				
1–2 members	2.68 [2.27–3.17]^***^	11.46 [8.89–14.79]^***^	1.00 [reference]	1.00 [reference]
3–5 members	1.96 [1.72–2.23]^***^	3.31 [2.83–3.87]^***^	1.42 [1.25–1.61]^***^	1.11 [0.93–1.32]
6–7 members	1.69 [1.46–1.96]^***^	2.15 [1.81–2.54]^***^	1.55 [1.34–1.79]^***^	1.09 [0.88–1.36]
8 or more	1.00 [reference]	1.00 [reference]	1.53 [1.30–1.79]^***^	1.03 [0.79–1.33]
**Household has child under-5**^**M**^				
No	1.00 [reference]	1.00 [reference]	1.00 [reference]	1.00 [reference]
Yes	0.85 [0.78–0.94]^**^	0.99 [0.87–1.12]	1.59 [1.46–1.74]^***^	1.40 [1.26–1.56]^***^
**Household has a pregnant woman**				
No	1.00 [reference]	–	1.00 [reference]	–
Yes	0.88 [0.74–1.05]	–	1.10 [0.93–1.31]	–
**Received net from PMD campaign**				
No	1.00[reference]	1.00[reference]	1.00 [reference]	1.00 [reference]
Yes	22.53[19.48–26.05]^***^	29.43[24.21–35.79]^***^	1.80 [1.52–2.13]^***^	1.00 [0.81–1.24]
**Number of nets in household**				
One	–	–	1.00 [reference]	1.00 [reference]
Two	–	–	1.45 [1.25–1.68]^***^	1.03 [0.86–1.24]
Three	–	–	1.58 [1.37–1.83]^***^	1.03 [0.84–1.27]
>Three	–	–	1.69 [1.48–1.94]^***^	1.00 [0.79–1.25]
**Universal coverage of LLINs**				
Not covered	–	–	1.00 [reference]	1.00 [reference]
Covered	–	–	1.22 [1.08–1.37]^**^	1.25 [1.06–1.48]^**^
**Respondents’ characteristics**				
**Respondent household**				
Household head	1.00 [reference]	1.00 [reference]	1.00 [reference]	–
Others	0.91 [0.83–1.00]^*^	0.98 [0.87–1.12]	0.95 [0.87–1.04]	–
**Respondent’s age**^**M**^				
18-49 years	1.00 [reference]	1.00 [reference]	1.00 [reference]	–
>49 years	1.13 [1.02–1.25]^*^	0.95 [0.82–1.10]	0.91 [0.83–1.01]	–
**Sex**				
Male	1.00 [reference]	-	1.00 [reference]	–
Female	1.06 [0.96–1.18]	-	0.92 [0.84, 1.03]	–
**Marital status**^**M**^				
Single	1.00 [reference]	1.00 [reference]	1.00 [reference]	1.00 [reference]
Divorced/separated	1.43 [1.16–1.76]^**^	1.27 [0.96–1.68]	1.21 [0.99–1.48]	1.10 [0.88–1.37]
Cohabiting	1.11 [0.84–1.46]	1.03 [0.73–1.46]	1.53 [1.16–2.02]^**^	1.14 [0.84–1.55]
Widowed	1.44 [1.20–1.74]^***^	1.00 [0.77–1.30]	1.14 [0.96–1.36]	1.09 [0.89–1.33]
Married	1.28 [1.13–1.45]^***^	1.23 [1.03–1.46]^*^	1.54 [1.36–1.75]^***^	1.31 [1.13–1.52]^***^
**Highest education**^**M**^				
No formal education	1.00 [reference]	1.00 [reference]	1.00 [reference]	1.00 [reference]
Primary	0.91 [0.79–1.05]	0.98 [0.82–1.17]	1.11 [0.97–1.27]	1.11 [0.95–1.29]
JHS/JSS/Middle	0.95 [0.85–1.06]	1.00 [0.86–1.15]	1.04 [0.94–1.16]	1.02 [0.90–1.16]
SHS/SSS/VOC/TECH	0.89 [0.76–1.04]	1.11 [0.90–1.36]	0.81 [0.69–0.93] **	0.93 [0.78–1.10]
Tertiary	0.67 [0.54–0.84]^***^	1.03 [0.76–1.41]	0.84 [0.67–1.07]	1.00 [0.77–1.30]
**Religion**^**M**^				
None	1.00 [reference]	–	1.52 [1.12–2.06]^**^	1.29 [0.92–1.79]
Christian	1.27 [0.97–1.66]	–	1.29 [1.14–1.46]^***^	1.35 [1.15–1.58]^***^
Muslim	1.18 [0.88–1.58]	–	1.00 [reference]	1.00 [reference]
Traditional	1.32 [0.93–1.88]	–	1.59 [1.24–2.05]^***^	1.26 [0.94–1.67]
**Employment status**^**M**^				
Unemployed	1.00 [reference]	–	1.00 [reference]	1.00 [reference]
Employed	1.05 [0.92–1.21]	–	1.25 [1.09–1.42]^**^	1.08 [0.93–1.24]
**Aware of benefits of LLINs**				
No	1.00 [reference]	1.00 [reference]	1.00 [reference]	1.00 [reference]
Yes	3.92 [3.57–4.32]^***^	1.09 [0.87–1.37]	1.89 [1.72–2.07]^***^	1.42 [1.18–1.71]^***^
**Aware of key facts of LLINs**				
No	1.00 [reference]	1.00 [reference]	1.00 [reference]	1.00 [reference]
Yes	4.05 [3.68–4.46]^***^	1.38 [1.11–1.72]^**^	1.78 [1.62–1.95]^***^	1.15 [0.96–1.39]
**Aware of continuous use and care for LLINs**				
No	1.00 [reference]	1.00 [reference]	1.00 [reference]	1.00 [reference]
Yes	1.94 [1.76–2.14]^***^	1.03 [0.90–1.19]	1.94 [1.77–2.12]^***^	1.08 [0.96–1.21]

COR, crude odds ratio; AOR, adjusted odds ratio; CI, confidence interval; *p*-value notation: ^*^p < 0.05, ^**^p < 0.01, and ^***^p < 0.001.

Universal coverage of LLINs was over 11 folds higher in households with 1–2 members (AOR: 11.46, 95% CI: 8.89–14.79), 3 folds higher in households with 3–5 members (AOR: 3.31, 95% CI: 2.83–3.87), and 2-folds higher in a 6–7members (AOR: 2.15, 95% CI: 1.81–2.54) as against households with 8 or more members. Those who received LLINs from PMD’s had a higher Universal coverage, about 30-fold higher as against those from different sources (AOR: 29.43, 95% CI: 24.21–35.79; Table 3).

Compared to respondents who were single, there was 23% increased odds of universal coverage of LLINs among respondents who were married (AOR: 1.23, 95% CI: 1.03–1.46). Universal coverage of LLINs was high among households whose representative was aware of the key facts of LLINs (AOR: 1.38, 95% CI: 1.11–1.72; Table 3).

### Multivariable analysis of factors associated with utilization of LLINs

From the adjusted logistic regression model, the odds of utilization of LLINs was higher among households in the Western (AOR: 2.59, 95% CI: 1.82–3.68), Ashanti (AOR: 2.19, 95% CI: 1.63–2.94), Eastern (AOR: 5.02, 95% CI: 3.68–6.86), Central (AOR: 2.90, 95% CI: 2.13–3.93), Volta (AOR: 6.49, 95% CI: 4.90–8.60), Brong Ahafo (AOR: 5.01, 95% CI: 3.79–6.63), and Northern (AOR: 3.52, 95% CI: 2.53–4.88) regions compared to households from the Greater Accra region. There was about 4-fold increase in household utilization of LLINs in rural areas compared to urban areas (AOR: 3.78, 95% CI: 2.73–5.24).

An interaction effect between residence (rural) and region of residence also showed increased utilization in the rural parts across all 9 regions: Greater Accra (AOR: 3.78, 95% CI: 2.73–5.24), Ashanti (AOR: 2.93, 95% CI: 2.32–3.70), Eastern (AOR: 8.96, 95% CI: 6.80–11.80), Central (AOR: 4.67, 95% CI: 3.51–6.23), Volta (AOR: 15.99, 95% CI: 11.48–22.25), Brong Ahafo (AOR: 5.97, 95% CI: 4.59–7.75), Western (AOR: 4.49, 95% CI: 3.46–5.82), Northern (AOR: 8.13, 95% CI: 6.05–10.92), and Upper East (2.15, 95% CI: 1.61–2.86; Table 3).

Utilization of LLINs was 40% higher in households with at least one child aged below 5 years (AOR: 1.40, 95% CI: 1.26–1.56) and 25% higher in households with universal access to LLINs (AOR: 1.25, 95% CI: 1.06–1.48; Table 3).

Additionally, compared to household representatives who were single, there was 31% increased odds of utilization of LLINs among respondents who were married (AOR: 1.31, 95% CI: 1.13–1.52). There were 35% increased odds of utilization of LLINs among respondents who were Christians (AOR: 1.35, 95% CI: 1.15–1.58) against Muslims (Table 3).

## Discussion

The purpose of this study was to evaluate the universal coverage and utilization of long-lasting insecticide nets across nine (9) out of the ten (10) regions of Ghana at the time of the study. Long-lasting insecticide nets are the core malaria prevention methods used across the globe by people who are at risk of malaria infection. In this study, 88% of households own at least one LLIN which is a higher percentage compared to 74%, 68%, and 48% recorded by the most recent Ghana Malaria Indicator Survey 2019 (15), Ghana Demographic and Health Survey (GDHS) 2014 (16) and Adjei and Gyimah in 2012 (17) respectively. However, there are other studies conducted across the world which recorded LLINs ownership similar to or above the current study (18–22). The high percentage of LLINs ownership could be attributed to the free mass distribution of LLINs programs coupled with the continuous distribution of LLINs in health facilities and schools, being undertaken in malaria endemic countries across the world including Ghana with increasing support from partners such as Global Fund, USAID, World Bank, US President’s Malaria Initiative (PMI) (23, 24).

A higher percentage of households living in rural than urban areas own LLINs—91% vs. 83%, which is consistent with the Ghana 2014 DHS findings, 78% vs. 60%, respectively, and the findings of other studies (25, 26). Universal coverage of LLIN differs significantly by geographic location in Ghana with more households in Upper East region owning LLINs. This finding is also consistent with a subnational profiling analysis of LLINs ownership and use conducted in Nigeria which revealed significant variation among ownerships in subnational locations (27). The heterogeneous transmission of malaria across Ghana could explain why the region of location may influence the universal coverage and use of LLINs. This is because of the high endemicity of malaria in the northern parts of Ghana and other areas in the middle belt during the raining season, more NGOs and some malaria research institutions operate in those areas. As a result, more social and behavior change communication messages are being broadcasted in those regions to create awareness about malaria prevention and the benefits of LLINs use.

The 80% universal coverage recommendation from WHO of providing each household with one LLIN for 2 people, was not achieved (28, 29). However, there has been a marked increase in universal coverage of LLINs since GDHS in 2014. This study showed that 76% of households in the study area have at least one LLIN for every two people who stayed in the household the night before the survey compared to 45% reported by the GDHS in 2014 (16) and 52% by the GMIS in 2019 (15). This is partly due to the increased availability of LLINs since 2010–2012 mass campaigns that saw a distribution of over 12 million LLINs (9); it is also due to increased awareness of the benefits of using LLINs (29, 30). Universal coverage was higher among households which received LLINs from the PMD campaign and households whose representatives were aware of the key facts of LLINs. This finding suggested that the goal of universal coverage in terms of the adequate provision of nets can only be achieved through sustained mass and continuous distribution systems and education activities. A similar conclusion was drawn in a study that evaluated a universal coverage campaign in Tanzania (31). Therefore, we recommend that the distribution exercise continue unabated.

In most studies including this current one, LLIN use rates fell below the WHO and Roll back malaria Partnership target of 80% (32, 33). With Ghana almost doubling its LLIN’s utilization from 2014 (36%) to the current study (66%), still much more improvement is needed. Studies have shown that countries such as Madagascar, Uganda, and Equatorial Guinea are doing better than Ghana in terms of LLIN use (19, 20). Households in rural areas are more likely than urban households to sleep under LLIN (70% vs. 57%) (25). Hence, an urban region like Greater Accra recorded 39% utilization compared to less urban like Volta region with 81% net utilization. This finding is in sync with most studies and therefore ignites the call for more awareness creation on the benefits of LLINs use, especially in peri-urban and urban slums (25, 26). Other studies, Ladi-Akinyemi et al. and Aung et al. have also reported the association of rural dwellers with LLIN ownership and use (25, 26). Perhaps this may be due to poor household structures that exist in rural areas hence the need to use the nets as a more effective extra layer against mosquito bites. In urban areas, household are more likely to have good structures which may prevent mosquitoes from entry. People living in urban areas may be able to use other alternative preventive measures such as mosquito spray and coils. On the contrary, a study found that urban households (72.1%) owned LLINs than rural (64.6%) ones (34). Though there is the perception that people living in urban settings may be able to purchase or use alternative approaches, concerted effort need to be made in other to reach out to people in these areas if the goal is to completely eradicate malaria in Ghana.

Utilization of LLINs was higher among households with children under 5 years. A similar study conducted in Nigeria also revealed that households with children under age 5 were more likely to sleep under an LLIN than other households with older children and adults (25). The finding that a household with at least one child aged below 5 years is more likely to use the LLIN compared to a household without a child under the age of five is very re-assuring. This means that, with the PMD program, vulnerable groups like children are more likely to be protected than older children and adults. Increased odds of households using LLIN was associated with children below age 5 in studies elsewhere in Kenya and Zimbabwe (35, 36). Households with smaller size (<8) were more likely to own bed nets compared to households with larger size. This may be due to an uneven distribution on the part of the field officers, probably as a result of the shortage of nets.

Furthermore, married respondents were 23% more likely to own a net when compared with single respondents. This is consistent with Kimbi et al. (37) who linked the finding to the fact that married women received financial aid from their husbands unlike single women who struggle on their own to take care of all family responsibilities (37). Findings from elsewhere reasoned that a household with both parents living together is more likely to make a better decision in favor of the use of LLINs to prevent malaria infection than others (33, 38).

Findings of the study further showed that the utilization of LLIN in households was directly associated with the household with one or more nets for every 2 persons. Long-lasting insecticide net (LLIN) use is ultimately the most important action needed for malaria control as people cannot use LLINs if they do not have access (39). Studies have shown that most rates of LLIN use among those with access to LLINs are at 80% target or above (40). Thus, increasing LLIN access will lead directly to increases in LLIN use. Awareness of the benefits of LLINs is another predictor of LLIN use found in this study. This finding is in harmony with studies conducted by Aina and Ayeni and Birhanu (33, 40). The 30-folds significance difference in universal coverage between households who received LLINs from PMD campaign and those who did not may be explained by the effectiveness of the PMD program to achieve sustained universal coverage of LLINs for malaria prevention (28, 29).

### Strength and limitations

The research assistants and field supervisors were well trained to carry out and ensure high-quality data collection process. The multistage sampling approach that was adopted ensured an adequate representation of the diverse population across regions and districts as well as urban and rural communities. In terms of limitations, there is the possibility of self-reporting bias as a result of respondents providing socially desirable responses. The second limitation is as a result of recall bias, which also has the potential to affect the results and the conclusions arrived at.

## Conclusion

Access, universal coverage, and utilization of LLINs continue to increase in Ghana, especially in the rural areas. The predictors of universal coverage of LLINs are region of residence, rural dwellers, household size of more than 2, receiving LLIN from PMD campaign, married, and awareness of key facts of LLINs. The predictors of utilization of LLINs are region of residence, rural dwellers, households with a child under age 5, households with universal coverage of LLIN, awareness of the benefits of LLINs, married, and Christianity.

## Data availability statement

The data analyzed in this study is subject to the following licenses/restrictions: the data has not been made publicly available. Requests to access these datasets should be directed to cbguure@ug.edu.gh.

## Ethics statement

The studies involving human participants were reviewed and approved by Ghana health Service Ethics Review Committee. The patients/participants provided their written informed consent to participate in this study.

## Author contributions

SA, CG, EK, KM, and NP conceptualized the study. SA, CG, YA, and EK carried out data duration. SA, CG, YA, and EK led the statistical analysis, interpretation, and drafting of the main manuscript with support from KM, DB, NP, and OA. SA, YA, EK, KM, DB, AP, OA, and CG contributed to the design, protocol implementation, manuscript drafting, identification of communities, and preparation of this manuscript. All authors contributed to the article and approved the submitted version.

## Conflict of interest

The authors declare that the research was conducted in the absence of any commercial or financial relationships that could be construed as a potential conflict of interest.

## Publisher’s note

All claims expressed in this article are solely those of the authors and do not necessarily represent those of their affiliated organizations, or those of the publisher, the editors and the reviewers. Any product that may be evaluated in this article, or claim that may be made by its manufacturer, is not guaranteed or endorsed by the publisher.

## References

[1] World Health Organization. World malaria report 2019. Geneva: World Health Organization (2019).

[2] World Health Organization. World malaria report 2020. Geneva: World Health Organization (2020).

[3] World Health Organization. World malaria report 2021. Geneva: World Health Organization (2021).

[4] National Malaria Control Programme. An epidemiological profile of malaria and its control in Ghana. Ghana: Ghana Health Service (2013).

[5] RBM. Roll Back Malaria 2005–2015. Geneva: World Health Organization (2005).

[6] Ghana Health Service. The health sector in Ghana: Facts and figures 2018. Ghana: Ghana Statistical Service (2019).

[7] CDC. PRESIDENT’S MALARIA INITIATIVE GHANA: Malaria operational plan FY 2018. Ghana: Ghana Statistical Service (2018).

[8] Paintain LS Awini E Addei S Kukula V Nikoi C Sarpong D. Evaluation of a universal long-lasting insecticidal net (LLIN) distribution campaign in Ghana: cost effectiveness of distribution and hang-up activities. Malar J. (2014) 13:1–113. doi: 10.1186/1475-2875-13-71, PMID: 24581249 PMC3944985

[9] NMCP. Ghana Malaria Control Programme Periodic Bulletin. Accra: Ghana National Malaria Control Programme (2017).

[10] Ghana National Malaria Control Programme. NetApp. (2018) Available at: https://www.netapp.com/.

[11] World Health Organization. Global technical strategy for malaria 2016–2030 Geneva: World Health Organization (2015).

[12] Nyavor KD Kweku M Agbemafle I Takramah W Norman I Tarkang E. Assessing the ownership, usage and knowledge of insecticide treated nets (ITNs) in malaria prevention in the Hohoe municipality, Ghana. Pan Afric Med J. (2017) 28:67. doi: 10.11604/pamj.2017.28.67.9934, PMID: 29255537 PMC5724734

[13] Ernst KC Erly S Adusei C Bell ML Kessie DK Biritwum-Nyarko A. Reported bed net ownership and use in social contacts is associated with uptake of bed nets for malaria prevention in pregnant women in Ghana. Malar J. (2017) 16:13. doi: 10.1186/s12936-016-1660-4, PMID: 28049471 PMC5210303

[14] Ghana Statistical Service (GSS) and ICF. Ghana malaria Indicator survey 2019. Accra, Ghana, and Rockville, Maryland, USA: GSS and ICF (2020).

[15] Ghana Statistical Service (GSS), Ghana Health Service (GHS), and ICF Macro. Ghana demographic and health survey 2014. Accra, Ghana: GSS, GHS, and ICF Macro (2014).

[16] Adjei JK Gyimah SO. Household bednet ownership and use in Ghana: implications for malaria control. Can Stud Popul. (2012) 39:15–30. doi: 10.25336/P6ZS44

[17] Tassew A Hopkins R Deressa W. Factors influencing the ownership and utilization of long-lasting insecticidal nets for malaria prevention in Ethiopia. Malar J. (2017) 16:262. doi: 10.1186/s12936-017-1907-8, PMID: 28668084 PMC5493859

[18] Nuwamanya S Kansiime N Aheebwe E Akatukwasa C Nabulo H Turyakira E. Utilization of long-lasting insecticide treated nets and parasitaemia at 6 months after a mass distribution exercise among households in Mbarara municipality, Uganda: a cross-sectional community based study. Malaria Res. Treat. (2018) 2018:1–10. doi: 10.1155/2018/4387506 PMC6093084 30155242

[19] Finlay AM Butts J Ranaivoharimina H Cotte AH Ramarosandratana B Rabarijaona H. Free mass distribution of long-lasting insecticidal nets lead to high levels of LLIN access and use in Madagascar, 2010: a cross-sectional observational study. PLoS One. (2017) 12:e0183936. doi: 10.1371/journal.pone.0183936, PMID: 28850631 PMC5574546

[20] García-Basteiro AL Schwabe C Aragon C Baltazar G Rehman AM Matias A. Determinants of bed net use in children under five and household bed net ownership on Bioko Island, Equatorial Guinea. Malar J. (2011) 10:179. doi: 10.1186/1475-2875-10-179, PMID: 21714859 PMC3146899

[21] de Beyl CZ Acosta A Monroe A Nyanor-Fosu F Ofori JK Asamoah O. Impact of a 15-month multi-channel continuous distribution pilot on ITN ownership and access in eastern region, Ghana. Malar J. (2018) 17:124. doi: 10.1186/s12936-018-2275-8, PMID: 29566678 PMC5863820

[22] Finlay AM Butts J Ranaivoharimina H Cotte AH Ramarosandratana B Rabarijaona H. Free mass distribution of long lasting insecticidal nets lead to high levels of LLIN access and use in Madagascar, 2010: a cross-sectional observational study. PLoS One. (2017) 12:e0183936. doi: 10.1371/journal.pone.0183936, PMID: 28850631 PMC5574546

[23] WHO. World malaria report 2014, vol. 23. Geneva: WHO (2018). 247 p.

[24] Ladi-Akinyemi TW Ladi-Akinyemi BO Olatona FA Oluwole FA. Ownership and utilization of long-lasting insecticide nets among caregivers of children under-5 years in Ogun state, Nigeria: a rural–urban comparison. J Clin Sci. (2018) 15:145. doi: 10.4103/jcls.jcls_30_18

[25] Aung T Wei C McFarland W Aung YK Khin HS. Ownership and use of insecticide-treated nets among people living in malaria endemic areas of eastern Myanmar. PLoS One. (2016) 11:e0162292. doi: 10.1371/journal.pone.0162292, PMID: 27618440 PMC5019368

[26] Andrada A Herrera S Inyang U Mohammed AB Uhomoibhi P Yé Y. A subnational profiling analysis reveals regional differences as the main predictor of ITN ownership and use in Nigeria. Malar J. (2019) 18:185. doi: 10.1186/s12936-019-2816-9, PMID: 31138216 PMC6540480

[27] World Health Organization. Achieving and maintaining universal coverage with long-lasting insecticidal nets for malaria control Geneva: World Health Organization (2017).

[28] NMCP-GH. 2017 annual report. Accra: National Malaria Control Programme—Ghana health service; (2018).

[29] Gakpey K Baffoe-Wilmot A Malm K Dadzie S Bart-Plange C. Strategies towards attainment of universal coverage of long-lasting insecticide treated nets (LLINs) distribution: experiences and lessons from Ghana. Parasit Vectors. (2016) 9:35. doi: 10.1186/s13071-016-1328-5, PMID: 26794136 PMC4722743

[30] West PA Protopopoff N Rowland MW Kirby MJ Oxborough RM Mosha FW. Evaluation of a national universal coverage campaign of long-lasting insecticidal nets in a rural district in north-West Tanzania. Malar J. (2012) 11:1–8. doi: 10.1186/1475-2875-11-273 22882836 PMC3465191

[31] Roll Back Malaria Partnership. The global malaria action plan for a malaria-free world. Director. (2008) 274:25–26.

[32] Seyoum D Speybroeck N Duchateau L Brandt P Rosas-Aguirre A. Long-lasting insecticide net ownership, access and use in Southwest Ethiopia: a community-based cross-sectional study. Int J Environ Res Public Health. (2017) 14:1312. doi: 10.3390/ijerph14111312, PMID: 29077052 PMC5707951

[33] Aina BA Ayeni FA. Knowledge and use of insecticide treated nets as a malaria preventive tool among pregnant women in a local government area of Lagos state, Nigeria. J Appl Pharmaceut Sci. (2011) 1:162.

[34] Zhou G Li JS Ototo EN Atieli HE Githeko AK Yan G. Evaluation of universal coverage of insecticide-treated nets in western Kenya: field surveys. Malar J. (2014) 13:351. doi: 10.1186/1475-2875-13-351, PMID: 25187326 PMC4162923

[35] Tapera O. Determinants of long-lasting insecticidal net ownership and utilization in malaria transmission regions: evidence from Zimbabwe demographic and health surveys. Malar J. (2019) 18:278. doi: 10.1186/s12936-019-2912-x, PMID: 31429761 PMC6701104

[36] Kimbi HK Nkesa SB Ndamukong-Nyanga JL Sumbele IU Atashili J Atanga MB. Socio-demographic factors influencing the ownership and utilization of insecticide-treated bed nets among malaria vulnerable groups in the Buea Health District, Cameroon. BMC Res Notes. (2014) 7:624. doi: 10.1186/1756-0500-7-624, PMID: 25204352 PMC4167508

[37] Bawo LL Harries AD Reid T Massaquoi M Jallah-Macauley R Jones JJ. Coverage and use of insecticide-treated bed nets in households with children aged under five years in Liberia. Public Health Action. (2012) 2:112–6. doi: 10.5588/pha.12.0040, PMID: 26392967 PMC4463064

[38] Koenker H Arnold F Ba F Cisse M Diouf L Eckert E. Assessing whether universal coverage with insecticide-treated nets has been achieved: is the right indicator being used? Malar J. (2018) 17:355. doi: 10.1186/s12936-018-2505-0, PMID: 30305127 PMC6180430

[39] Koenker H Kilian A. Recalculating the net use gap: a multi-country comparison of ITN use versus ITN access. PLoS One. (2014) 9:e97496. doi: 10.1371/journal.pone.0097496, PMID: 24848768 PMC4030003

[40] Birhanu Z Yihdego YY Yewhalaw D. Caretakers’ understanding of malaria, use of insecticide treated net and care seeking-behavior for febrile illness of their children in Ethiopia. BMC Infect Dis. (2017) 17:629. doi: 10.1186/s12879-017-2731-z, PMID: 28923020 PMC5604495

